# *Leishmania* Resistance to Miltefosine Associated with Genetic Marker

**DOI:** 10.3201/eid1804.110841

**Published:** 2012-04

**Authors:** Sandrine Cojean, Sandrine Houzé, Djamel Haouchine, Françoise Huteau, Sylvie Lariven, Véronique Hubert, Florence Michard, Christian Bories, Francine Pratlong, Jacques Le Bras, Philippe Marie Loiseau, Sophie Matheron

**Affiliations:** University of Paris-Sud, Châtenay-Malabry, France (S. Cojean, F. Huteau, C. Bories, P.M. Loiseau);; Paris Descartes University, Paris, France (S. Houzé, D. Haouchine, V. Hubert, J. Le Bras);; Paris Diderot University, Paris (S. Lariven, F. Michard, S. Matheron);; Montpellier University, Montpellier, France (F. Pratlong)

**Keywords:** Leishmania resistance, leishmaniasis, miltefosine, LdMT, *L. donovani*, antiretroviral, parasites

**To the Editor:** During 2000–2010, serial *Leishmania* isolates obtained from an HIV-infected patient who was not responding to treatment showed a gradual decrease in in vitro miltefosine susceptibility. We performed *L. donovani* miltefosine transporter (*Ldmt*) gene analysis to identify an association between miltefosine resistance of reference *L. donovani* lines and variability in miltefosine response of *L. infantum* isolates. A new single-nucleotide polymorphism (SNP), L832F, was identified, which might be a marker of miltefosine resistance in leishmaniasis.

The patient, a 46-year-old woman, had lived in France since 1994 but regularly returned to Algeria, her country of birth. HIV-1 infection was diagnosed in 1991. Antiretroviral therapy was initiated in 1993, leading to undetectable viral load and a CD4+ T-cell count of 185 cells/mm^3^ (reference >450/mm^3^). Concurrent conditions were thoracic herpes zoster in 1996, hairy leukoplakia of tongue, oropharyngeal candidiasis, and chronic renal failure of unknown cause since 2000.

Visceral leishmaniasis was diagnosed in 1998 by culture of a bone marrow smear, which showed intracellular amastigotes. Use of meglumine antimonate (Glucantime; Sanofi, Paris, France), a drug of choice for the treatment of leishmaniasis, was contraindicated because of pancreatitis in the patient and in vitro isolate susceptibility variation; therefore, induction therapy consisted of liposomal amphotericin B (AmpB [AmBisome; Astellas Pharma US, Deerfield, IL, USA]) at a dose of 3 mg/kg/d for 5 consecutive days, then 1× week for 5 weeks (total dose 30 mg/kg) during 1998–2000 (Table). The same medication was administered for relapses at 4 mg/kg/d for 5 days, then 4 mg/kg 1× week for 5 weeks (total dose 40 mg/kg) during 2001–2010. Given the adverse effects of AmpB and the availability of oral miltefosine (Impavido; AEterna Zentaris Inc., Quebec City, Quebec, Canada), the latter drug was used for maintenance treatment during 2001–2007 at 50 mg 2×/d. Leishmaniasis was monitored by leukocytoconcentration and culture of blood samples on Novy-Nicolle-McNeal medium.

**Table:** Comparions of IC_50_ for AmpB and miltefosine against promastigotes and axenic amastigotes and distribution of *LdMT* SNPs in *Leishmania infantum* isolates and reference strains*

Isolate	Year	AmpB	Miltefosine	IC_50_, µmol/L ± SEM					*Ldmt* SNP
				AmpB			Miltefosine		
				Promastigotes	Axenic amastigotes		Promastigotes	Axenic amastigotes	
–	1998	3 mg/kg/d × 5 d; then 1×/wk × 5 wk	–	–	–		–	–	–
S_1_	2000	3 mg/kg/d × 5 d; then 1×/wk × 5 wk	–	0.09 ± 0.04†	0.10 ± 0.03		7.14 ± 0.56†	5.00± ± 0.7†	L832
–	2001	4 mg/kg/d × 5 d; then 1×/wk × 5 wk	50 mg 2×/d	–	–		–	–	–
S_3_	2005	4 mg/kg/d × 5 d; then 1×/wk × 5 wk‡		0.13 ± 0.03	0.20 ± 0.03		25.93 ± 1.46†	21.00 ± 1.50†	832_L_/F
S_4_				0.24 ± 0.01†	0.15 ± 0.02		27.89 ± 1.76†	31.90 ± 1.60†	
–	2007	4 mg/kg/d × 5 d; then 1×/wk × 5 wk		–	–		–	–	–
S_6_	2008	4 mg/kg/d × 5 d; then 1×/wk × 5 wk	–	0.16 ± 0.03	0.11 ± 0.03		44.30 ± 3.70†	50.10 ± 1.00†	832F
S_7_	2010	4 mg/kg/d × 5 d; then 1×/wk × 5 wk	–	–	–		–	–	L832
Reference strain									
LV9 WT				0.03 ± 0.02	0.02 ± 0.05		4.46 ± 0.29†	6.20 ± 0.3	L832
LV9 Miltefosine-R				0.22 ± 0.04	0.70 ± 0.09		45.84 ± 2.40†	54.20 ± 2.20†	832F
DD8 WT				0.06 ± 0.02†	0.05 ± 0.03†		17.40 ± 1.70	12.40 ± 1.50	L832
DD8 AmpB-R				1.42 ± 0.06†	1.00 ± 0.07†		15.20 ± 1.00	10.30 ± 1.20	L832

*IC_50_, 50% inhibitory concentration; AmpB, amphotericin B; *Ldmt*, *Leishmania donovani* miltefosine transporter gene; SNP, single-nucleotide polymorphism; –, assay not performed because sample unavailable or not culturable; WT, wild type; R, resistant. †Significance was analyzed by using the nonparametric Mann-Whitney U test to compare the IC_50_ of the isolates with the IC_50_ of reference strains; p<0.01 was considered significant. IC_50_ of AmpB and miltefosine was compared with IC_50_ of reference strains and S_1_/S_3_, S_1_/S_4_, and S_1_/S_6_. Miltefosine: S_1_/S_3_, S_1_/S_4_, S_1_/S_6_, S_4_/S_6_; p<0.01. AmpB: S_1_/S_4_ significant p<0.01; S_1_/S_3_, S_1_/S_6_ not significant. ‡For each relapse.

When signs of biological and clinical relapse appeared, bone marrow was aspirated for parasite detection. After culture of the aspirate and isoenzyme determination, the strain was identified as *L. infantum*, zymodeme MON-24. Eleven relapses were documented; all were confirmed by positive direct examination of bone marrow or blood, but cultures of only 7 samples yielded positive results (Table).

The susceptibility of 4 cryopreserved isolates (S_1_, S_3_, S_4_, and S_6_; Table) to AmpB and to miltefosine was studied in the in vitro promastigote and axenic amastigote form by determining the concentrations inhibiting parasite growth by 50% (*1*,*2*). The 50% inhibitory concentration (IC_50_) was determined in parallel for the following reference *L. donovani* lines: a wild-type *L. donovani* LV9 (MHOM/ET/67/HU3) line (LV9 WT), a wild-type *L. donovani* DD8 (MHOM/IN/80/DD8) line (DD8 WT), a laboratory miltefosine-resistant line obtained from LV9 WT (LV9 miltefosine-R, resistant to 90 μmol/L miltefosine), and the laboratory AmB-resistant line obtained from DD8 WT (DD8 AmB-R, resistant to 1.4 μmol/L AmB) on promastigote and axenic amastigote forms (*3*,*4*).

The AmB susceptibility of the isolates did not change notably over time; IC_50_ values ranged from 0.09 µmol/L to 0.24 µmol/L, regardless of parasite form, similar to those of wild-type reference strains (Table). In contrast, the IC_50_ values of miltefosine increased greatly over time, from 5.00 µmol/L to 50.10 μmol/L. During the 6 years of follow-up with miltefosine maintenance therapy, the susceptibility of the isolate (S_6_) obtained 6 months after miltefosine treatment withdrawal in 2008 was 6-fold higher than that of the first isolate (S_1_) obtained in 2000.

The *L. donovani* miltefosine transporter protein (LdMT) promotes miltefosine translocation (*5*), and LdMT inactivation in *L. donovani* promastigotes leads to miltefosine resistance at the promastigote and amastigote stages (*6*). In 2003 and 2006 studies, several mutations were linked to the inability of parasites to take up miltefosine and to miltefosine resistance (*5*,*7*). In a 2009 study, the weak expression of LdMT and its β subunit LdROS3 in *L. braziliensis* isolates was linked to diminished sensitivity (*8*). We sequenced the entire *Ldmt* gene (3,294 bp) in the reference strains and the clinical isolates for SNP analysis (*5*,*7*). Only 1 new SNP, L832F, was found in the miltefosine-resistant reference strain (LV9 miltefosine-R) and in clinical isolate S_6_. The L832 wild-type allele was found in isolate S_1_ and in the miltefosine-sensitive reference lines (LV9, DD8, and DD8 AmpB-R), whereas both alleles were found in isolates S_3_ and S_4_, with a decrease in the wild-type allele (Table). The last isolate, which was obtained 3 years after miltefosine withdrawal and could not be subcultured, had reverted to the wild-type allele (L832).

These results point to a relation between the 832F allele and diminished susceptibility to miltefosine. Analysis of this case of miltefosine resistance in a patient co-infected with *Leishmania* sp. and HIV strongly suggests that an SNP (L832F) in the *Ldmt* gene could represent a molecular marker of miltefosine resistance in *L. infantum* and *L. donovani*.
